# Editorial: *Ex vivo* graft preservation and modification

**DOI:** 10.3389/frtra.2023.1291543

**Published:** 2023-10-23

**Authors:** David Al-Adra, Constanca Figueiredo, Nicco Krezdorn

**Affiliations:** ^1^ Division of Transplantation, Department of Surgery, University of Wisconsin School of Medicine and Public Health, Madison, WI, United States; ^2^ Institute for Transfusion Medicine, Hannover Medical School, Hanover, Germany; ^3^ Clinic for Plastic, Aesthetic, Hand and Reconstructive Surgery, Center for Surgery, Hannover Medical School, Hanover, Germany

**Keywords:** *ex-vivo*, transplantation, organ preservation and perfusion, organ modification, organ donation

**Editorial on the Research Topic** *Ex-vivo* graft preservation and modification

Organ transplantation is the best treatment for end-stage organ disease. However, the demand for suitable organs for transplant outweighs the supply, which has led to the expansion of the donor pool using marginal quality organs (1). With respect to kidneys, marginal organs may carry higher risks, but they have been shown to be better for patients than dialysis (2, 3). To mitigate the risks associated with marginal organs *ex vivo* machine perfusion has been implemented for a variety of solid organs (4–6). Variations on *ex vivo* perfusion technology have extended donor organ cold ischemia time, allowed for organ assessment, and opened the possibility of organ rehabilitation with drug-, cell-, or gene-therapy. As such, these perfusion platforms may allow therapies for the reduction of ischemia-reperfusion injury and attenuation of allo- or xenogeneic immunogenicity. Therefore, this Research Topic aims to showcase state-of-the-art techniques in *ex vivo* organ preservation and modification.

Vargas et al. begin the Research Topic with a review summarizing the rapidly advancing field of *ex vivo* perfusion technology with respect to the liver. This review discusses immunomodulation, gene therapy and pharmacotherapy to modify a liver graft prior to transplant using *ex vivo* perfusion. Although organ preservation via *ex vivo* perfusion systems is promising, this manuscript concludes by reflecting on the many steps still to overcome prior to the translation of these experimental *ex vivo* techniques to the clinical setting. One such clinical obstacle is determining the optimal oxygen carrier to supply adequate tissue oxygenation to meet the physiological metabolic demand of *ex vivo* perfusion when conducted at normothermic temperatures. To this end, Rother et al. studied the effects of three different oxygen carriers on porcine kidneys undergoing normothermic *ex vivo* perfusion. Using the Kidney Assist® device, the authors show similar kidney tissue integrity between oxygen carriers. However, using real-time polymerase chain reaction and Luminex techniques, the authors also show lower immunogenicity of the kidney after perfusion with all synthetic oxygen carriers vs. red blood cells. Similar to our recent findings in the liver, these results suggest alternative oxygen carriers to red blood cells are effective, may be more available, and have immunological advantages over packed red blood cells (7). Furthermore, Hofmann et al. presented an overview on the potential of using human amniotic membrane (hAM)-derivatives in decreasing immunogenicity and supporting tissue regeneration.

In addition to prolonging organ storage and offering functional assessment, one of the most exciting aspects of *ex vivo* perfusion is the unique advantage of being able to deliver therapeutics directly to the organ. However, not all *ex vivo* modifications require perfusion. In the study by Gao et al. recombinant adeno-associated virus (rAAV) is used to transduce rat livers with the firefly luciferase gene while undergoing cold storage. After syngeneic liver transplantation, control animals demonstrated no bioluminescent activity, while animals receiving rAAV-treated livers demonstrated gene expression based on bioluminescence and quantitative polymerase chain reaction. Taken together, these results show the effectiveness of rAAV mediated gene transduction in liver grafts when administered during cold storage.

Contained withing this Research Topic, we have demonstrated the latest techniques of *ex vivo* graft preservation and modification. Future studies will continue to expand the use of *ex vivo* perfusion and modification to expand the use of marginal organs for transplantation.
